# Insights Into Ocular Resilience: Unraveling the Epidemiology, Prognostic Factors, and Visual Triumphs of Open Globe Injuries in the COVID-19 Era at a Leading Tertiary Care Centre of Western Rajasthan, India

**DOI:** 10.7759/cureus.53198

**Published:** 2024-01-29

**Authors:** Seema Meena, Kavita Bhatnagar, Kirti Jaisingh, Jyoti Shakrawal, Manjari Tandon, Nikhil Agrawal

**Affiliations:** 1 Ophthalmology, All India Institute of Medical Sciences, Jodhpur, Jodhpur, IND

**Keywords:** ocular trauma score (ots), visual outcome, open globe injuries, pronostic factors, epidemiology of ocular trauma

## Abstract

Introduction: This study was conducted to describe the epidemiology, prognostic factors, and visual outcomes of open globe injuries (OGIs) at a tertiary care centre in Western Rajasthan, India.

Methods: Data of OGI patients treated at the Department of Ophthalmology, All India Institute of Medical Sciences, Jodhpur, Rajasthan, from March 2019 to December 2021 were reviewed retrospectively. Demographic details including age, gender, place of injury, wound characteristics (i.e., cause, mechanism, location, and size), visual acuity (VA), and associated ocular injuries were recorded. The Ocular Trauma Classification System (OTCS) and the Birmingham Eye Trauma Terminology (BETT) were used to categorize OGIs. All the data was entered into a Microsoft Excel sheet (Microsoft Corporation, Redmond, Washington, United States) and analyzed with IBM SPSS Statistics for Windows, Version 23.0 (Released 2015; IBM Corp., Armonk, New York, United States).

Results: A total of 40 patients with OGIs were included. OGI was discovered to be four times higher in males and 47.5% of the total cases were observed in patients aged 0-15 years, followed by patients aged 16-30 (27.5%). The rupture injury showed a higher incidence rate (32.5%) than the penetrating injury (62.5%). Of all OGIs, 75% were grade 4 injuries, with zone 1 being the most often affected zone and wood stick injury being the most prevalent aetiology. A significant difference was noted (p<0.001) in comparing presenting VA with final VA with paired sample t-test. A negative Spearman correlation was noted between age and final VA (r = 0.53, p = 0.000), and the grade of injury and final VA (r = 0.51, p = 0.001.)

Conclusion: Young males were the most susceptible group to sustain OGIs due to their more physical activities. Health education and safety in the workplace and during sports are crucial to reduce the incidence of OGI.

## Introduction

Ocular trauma stands as a significant contributor to ocular morbidity, forming a substantial portion of emergency consultations. According to the World Health Organization, approximately 55 million cases of ocular trauma result in restricted activities for over one day each year [1]. The gravity of the issue is further underscored by the annual occurrence of 1.6 million cases of binocular blindness and 19 million cases of monocular blindness due to eye trauma [2,3]. Disparities in the incidence of blindness from eye trauma exist between developed and developing countries, with 0.9 cases per lakh people in developed nations compared to 75 per lakh people in developing regions [4,5]. Among ocular trauma cases, open globe injuries (OGI) pose a higher degree of severity and vision-threatening consequences when compared to closed globe injuries [6]. However, timely surgical intervention and visual rehabilitation play pivotal roles in improving the prognosis of OGI cases [6,7].

OGI is characterized by a complete thickness break or rupture of the cornea, sclera, or both, constituting 10% of all ocular injuries. Prognostic factors play a crucial role in determining the success of interventions [7,8]. Notably, Kuhn et al. introduced the ocular trauma score, a simplified prognosis predictor model assigning numerical points to variables such as globe rupture, perforating injury, initial visual acuity (VA), retinal detachment, endophthalmitis, and relative afferent pupillary defect (RAPD) [8].

The emergence of the coronavirus disease 2019 (COVID-19) virus in 2019 significantly influenced human social behavior, leading to widespread social isolation and the adoption of the 'do-it-yourself' approach [9]. Telemedicine has become the primary means of connecting with healthcare professionals, resulting in a shift in healthcare-seeking behavior. Studies conducted during this period have indicated a decline in ocular trauma-related emergencies and have shed light on changing patterns in injury incidences [10].

Despite the global impact of OGI, there is a notable scarcity of studies comprehensively investigating the demographic, clinical, and prognostic profiles of these injuries. Therefore, our study aims to retrospectively evaluate the epidemiology, patterns, prognostic factors, and outcomes of OGI at a tertiary care center in Western India during the COVID-19 era.

## Materials and methods

This retrospective analysis was conducted by the Department of Ophthalmology at the All India Institute of Medical Sciences (AIIMS), Jodhpur, Rajasthan, and aimed to comprehensively investigate the outcomes of OGI repair procedures performed between March 2019 and December 2021. This study was meticulously designed and executed, adhering to ethical guidelines outlined in the Declaration of Helsinki, and received formal approval from the Institutional Ethics Committee, AIIMS, Jodhpur (reference number: AIIMS/IEC/2022/3911).

To ensure the robustness of the study, stringent inclusion and exclusion criteria were applied. Inclusion criteria focused on patients who underwent OGI repair at AIIMS, Jodhpur, during the stipulated period, with a minimum three-month postoperative follow-up and complete medical records. Patients with a history of OGI repair from external institutions or those with incomplete records were excluded to maintain data integrity.

The data collection process involved a meticulous review of medical records, extracting key demographic details such as age, gender, and location of injury. The examination of wound characteristics delved into the cause, mechanism, location, and size of the OGI. VA was systematically documented at the presentation and at the final follow-up. Furthermore, associated ocular findings, including lid tear, hyphema, iris prolapse, lens status, vitreous hemorrhage (VH), intraocular foreign body (IOFB), retinal detachment, and endophthalmitis, were comprehensively assessed.

To enhance the precision and consistency of the analysis, two established classification systems, the Birmingham Eye Trauma Terminology (BETT) and Ocular Trauma Classification System (OTCS), were employed. This classification framework included categorization based on the type of injury (rupture, penetration, IOFB, perforation, and mixed), the wound's anatomical zone (Zone I, Zone II, and Zone III), VA grade (Grade A to Grade E), and pupil status (positive RAPD, negative RAPD).

Outcome measures were carefully defined, with the final best-corrected VA (BCVA) serving as a pivotal indicator. A BCVA of less than or equal to 4/200 (grades 4 and 5) was considered indicative of a poor visual outcome. The data management process involved the use of Microsoft Excel (Microsoft Corporation, Redmond, Washington, United States) for meticulous organization, and statistical analysis was conducted using IBM SPSS Statistics for Windows, Version 23.0 (Released 2015; IBM Corp., Armonk, New York, United States). This comprehensive methodology ensures a thorough examination of OGI repair outcomes, contributing nuanced insights to the landscape of ophthalmic research and clinical practice.

## Results

The study encompassed a comprehensive analysis of 40 cases of OGIs. Within this cohort, a notable male predominance was observed, with a male-to-female ratio of 4:1, signifying a higher incidence of such injuries among males (Table 1). Geographically, 60% (n=24) of the cases originated from urban areas, while the remaining cases were reported from rural regions, reflecting a degree of urban-rural variation in the occurrence of OGIs. Age-wise distribution revealed that the age group of 0-15 years was the most vulnerable age group, comprising a substantial 47.5% of the cases. This suggests that children and adolescents are particularly at risk for such ocular trauma. The age group of 16-30 years accounted for 27.5% (n=19) of cases, indicating that young adults also face a significant incidence of OGIs. The 31-45 years age group contributed 15% (n=6) of the cases, and a smaller proportion, 10% (n=4), was observed in individuals older than 45 years. In terms of laterality, left eye injuries predominated, constituting 55% (n=22) of the cases, whereas right eye injuries comprised the remaining 45% (n=18).

**Table 1:** Demographic profile of the participants

Demographic charateristics	Frequency (percentage)
Age	
0-15	19 (47.5%)
16-30	11 (27.5%)
31-45	6 (15%)
>45	4 (10%)
Sex	
Males	32 (80%)
Females	8 (20%)
Location	
Rural	16 (40%)
Urban	24 (60%)
Eye	
Right	18 (45%)
Left	22 (55%)

The predominant type of injury was penetrating, constituting the highest incidence at 62.5% (n=25), indicating a substantial prevalence of sharp objects causing ocular damage. Rupture, representing a distinct mode of injury, followed closely at 32.5% (n=13). Remarkably, there were no instances of perforating or mixed injuries, underscoring the specificity of the identified cases. Intriguingly, IOFB was identified in 5% of cases (n=2), shedding light on the diverse nature of penetrating injuries within the cohort. This finding emphasizes the need for meticulous examination and imaging to detect and manage such ocular foreign bodies, considering their potential implications for visual outcomes.

The etiological panorama elucidated the multifaceted origins of OGIs, with wooden stick injuries emerging as the most prevalent, accounting for 35% of cases (n=14) (Table 2). Road traffic accidents, a significant contributor, followed at 12.5% (n=5), highlighting the impact of external forces in urban settings. Hammer and chisel injuries, representing occupational hazards, were notable at 10% (n=4). Wire and other iron objects, iron rod injuries, firecracker injuries, stone injuries, pen injuries, glass injuries, cow horn injuries, and scissor injuries collectively contributed to the intricate tapestry of ocular trauma within the study group.

**Table 2:** Clinical profile of patients

Clinical characteristics	Frequency (percentage)
Mode of Injury	
Wooden stick	14 (35%)
Iron rod	2 (5%)
Road traffic accident	5 (12.5%)
Cow horn	1 (2.5%)
Hammer and chisel	4 (10%)
Glass	1 (2.5%)
Firecracker	2 (5%)
Wire	3 (7.5%)
Stone	2 (5%)
Scissor	1 (2.5%)
Pen	2 (5%)
Other iron body	3 (7.5%)
Zone of injury	
Zone 1	31 (77.5%)
Zone 2	7 (17.5%)
Zone 3	2 (5%)
Grade of injury	
Grade 1	1 (2.5%)
Grade 2	3 (7.5%)
Grade 3	2 (5%)
Grade 4	30 (75%)
Grade 5	4 (10%)
Others	
Non-reacting pupil	22 (55%)
Hyphaema	13 (32.5%)
Iris prolapse	33 (82.5%)
Cataract	9 (22.5%)
Dislocated lens	1 (2.5%)
Intraocular foreign body	3 (7.5%)
Vitreous hemorrhage	3 (7.5%)
Retinal detachment	1 (2.5%)
Endophthalmitis	1 (2.5%)
Lid tear	4 (10%)

Anatomically, the distribution of injuries across different zones revealed Zone 1 as the most common, accounting for a substantial 77.5% (n=31). Zone 2 and Zone 3 injuries followed at 17.5% (n=7) and 5% (n=2), respectively. This detailed categorization provides a nuanced understanding of the varying extents of ocular trauma, enabling clinicians to tailor interventions based on injury severity and location. Examination of pupil status brought to light that 45% of patients exhibited RAPD in the injured eye, indicating compromised optic nerve function. The grading of injuries, ranging from Grade 1 to Grade 5, further elucidated the severity spectrum. Lid tear, a distinctive feature, was present in 10% of cases (n=4), signifying additional challenges in the management of these injuries. The anterior segment findings illuminated the varied ocular presentations, with iris prolapse (82.5%; n=33), hyphema (32.5%; n=13), and vitreous prolapse (5%; n=2) offering insights into the complexity of these injuries. Cataracts, observed in 22.5% of cases (n=9), underscored the secondary lens-related complications in the aftermath of open globe trauma. Posterior segment manifestations included VH (7.5%; n=3), IOFB (7.5%; n=3), retinal detachment (2.5%), and endophthalmitis (2.5%; n=1), providing a comprehensive view of the diverse structural implications affecting the posterior eye.

The therapeutic approach was uniformly directed towards primary globe perforation repair in all cases, emphasizing the urgency and necessity of immediate surgical intervention. However, secondary surgery was deemed necessary in 20% of cases (n=8), addressing complications such as traumatic cataract, subluxated lens, VH, nucleus/IOL drop, retinal detachment, IOFB, and endophthalmitis. The culmination of these clinical endeavors resulted in a spectrum of final VA outcomes. Notably, 40% of cases (n=16) achieved a VA of 6/60 or better, showcasing the efficacy of interventions in a significant proportion. However, a considerable 60% of cases (n=24) exhibited VA less than 6/60, indicating the varied and challenging nature of visual rehabilitation in OGIs. Perhaps most strikingly, 10% of cases (n=4) showed no perception of light (PL) vision, emphasizing the profound impact of these injuries on visual function (Table 3).

**Table 3:** Final best corrected visual acuity of patients PL: perception of light

Characteristics	Frequency (percentage)
No PL	4 (10%)
PL+ to 5/60	20 (50%)
≥6/60	16 (40%)

Statistical analyses, including a paired sample t-test, revealed a significant difference between presenting VA and final VA (p < 0.001, 95%CI -2.50 to -1.00), providing quantitative support to the observed clinical outcomes. Moreover, negative Spearman's correlation coefficients were noted between age and final VA (r = 0.53, p = 0.000), as well as between the grade of injury and final VA (r = 0.51, p = 0.001), offering valuable insights into the factors influencing visual outcomes in OGIs.

The study highlighted a significant disparity between the initial and concluding VA (p < 0.001, 95%CI -2.50 to -1.00), substantiated by a paired sample t-test, indicating a marked enhancement in visual outcomes post intervention. Additionally, Spearman's correlation analysis revealed a negative correlation between age and final VA (r = 0.53, p = 0.000), implying a potential decline in ultimate visual outcomes with increasing age. Similarly, a negative correlation was noted between the grade of injury and final VA (r = 0.51, p = 0.001), underlining the connection between injury severity and poorer final visual outcomes. These statistical findings provide valuable insights into the impact of age and injury severity on the ultimate VA in individuals with OGIs.

## Discussion

With the onset of the COVID-19 pandemic, stringent restrictions were enforced, leading to a new societal norm of social distancing and limited gatherings at festivities, weddings, and other celebrations. Consequently, there has been a decline in global cases of ocular trauma, reshaping the injury landscape. However, the documented increase in instances of domestic violence is noteworthy, as indicated by a few studies [11]. Our study evaluated the visual outcomes after OGIs and changing trends in demography and mode of injury in the time of COVID-19 in the Indian population. 

The current study reaffirmed the predominance of males, consistent with earlier research that has linked this trend to the active engagement of males in risky occupational settings and outdoor pursuits [12]. Importantly, it is worth noting that a substantial portion of occupational injuries, estimated at approximately 90%, are preventable [13]. While previous investigations have typically reported a higher incidence of ocular injuries within the age group of 18-50 years [13-16], a noteworthy observation in our study was the significant prevalence of OGIs among children under the age of 18. This observation suggests that a potential lack of awareness among caregivers could be a primary contributing factor to this variation.

Penetrating injury emerged as the most common mechanism in the present study, followed by globe rupture. Wooden stick injury, iron nails, and wire were major contributors to over half of the OGIs, with road traffic accidents and chisel and hammer injuries following.

Studies by other authors employing univariate and multivariate analyses emphasized the pivotal role of initial VA as the most significant predictive factor in a patient's visual rehabilitation [16,17]. This aligns with our study, where VA at presentation was identified as the major prognostic factor for favorable final VA. Injuries associated with VH, retinal detachment, and IOFB were linked to poor visual outcomes. However, encouragingly, post-traumatic cataract patients exhibited significant vision improvement after cataract surgery and IOL implantation.

Despite our study being conducted during the COVID-19 era, the results and outcomes closely resembled those of studies conducted in the pre-COVID-19 era. While a prior study by Halawa et al. reported an increase in injuries at home during the pandemic [17], the current study indicated that occupational injuries remained the primary cause. This could be attributed to the return to near-normalcy over two years, compared to the initial phases of the COVID-19 pandemic when home or institutional confinement was enforced by the government. It is important to note the limitations of our study, including a small sample size and its retrospective nature, which adds to its disadvantages.

## Conclusions

Our retrospective analysis underscores the critical role of VA at the time of presentation as the major prognostic factor influencing favorable final visual outcomes in cases of OGIs. Notably, injuries associated with VH, retinal detachment, and IOFB were identified as significant contributors to poor visual outcomes. This emphasizes the importance of early recognition and intervention in cases presenting with these complications to optimize visual prognosis.

Moreover, the considerable proportion of impaired final VA highlighted in our study signals potential socioeconomic implications. Visual impairment can significantly impact an individual's ability to perform daily activities and may lead to economic consequences both at the individual and societal levels. Therefore, there is a pressing need for comprehensive health education initiatives and workplace safety promotion. These measures are crucial to reduce the occurrence of ocular injuries, particularly in occupational settings, and to enhance overall eye health. By fostering awareness and implementing preventive strategies, we can contribute to minimizing the burden of OGIs and improving the overall visual well-being of the population.
